# An overview of PAX1: Expression, function and regulation in development and diseases

**DOI:** 10.3389/fcell.2022.1051102

**Published:** 2022-10-28

**Authors:** Weiyin Wu, Xiangjun Kong, Yanhan Jia, Yihui Jia, Weimei Ou, Cuilian Dai, Gang Li, Rui Gao

**Affiliations:** ^1^ Institute of Cardiovascular Diseases, Xiamen Cardiovascular Hospital, School of medicine, Xiamen University, Xiamen, China; ^2^ Department of Pharmacy, Xiang'an Hospital of Xiamen University, School of medicine, Xiamen University, Xiamen, China; ^3^ Sichuan Cancer Hospital and Institute, Sichuan Cancer Center, School of Medicine, University of Electronic Science and Technology of China, Chengdu, China

**Keywords:** Pax1, transcription factor, embryonic development, diseases, cancer

## Abstract

Transcription factors play multifaceted roles in embryonic development and diseases. PAX1, a paired-box transcription factor, has been elucidated to play key roles in multiple tissues during embryonic development by extensive studies. Recently, an emerging role of PAX1 in cancers was clarified. Herein, we summarize the expression and functions of PAX1 in skeletal system and thymus development, as well as cancer biology and outline its cellular and molecular modes of action and the association of PAX1 mutation or dysregulation with human diseases, thus providing insights for the molecular basis of congenital diseases and cancers.

## 1 Introduction

Embryonic development is under tight regulation of transcription factors, dysfunction of which could lead to congenital defects, or even early embryonic lethality. Thus, revealing the roles of key transcription factors not only helps us to better understand normal embryonic development, but also the etiology of birth defects.

PAX1 is a transcription factor, playing crucial roles in diverse biological processes. It belongs to the paired box-containing (PAX) gene family which is highly conserved in both vertebrates and invertebrates (Thompson et al., 2021). Pax1 was initially identified from mouse in 1988 which showed highly homology to all the three paired-box genes isolated from *Drosophila* in 1986, called *paired* (*prd*), *gooseberry proximal* (*gsb-p*) and *gooseberry distal* (*gsb-d*) (Deutsch et al., 1988).

**FIGURE 1:**
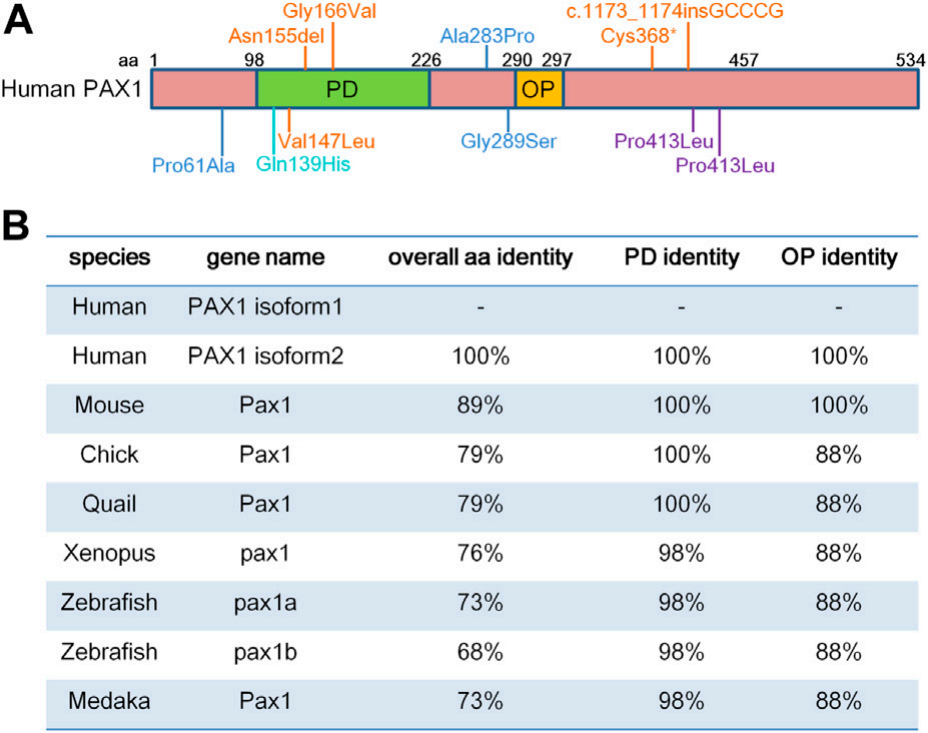
PAX1 is a highly conserved transcription factor. **(A)** Schematic representation of human PAX1 protein domains. It contains a highly conserved paired-box domain (PD) and an octopeptide domain (OP). Amino acid (aa) positions are indicated above. Locations of PAX1 mutations identified in human diseases are also marked. **(B)** Identity in the overall amino acid sequence, PD and OP domains between human PAX1 and PAX1 proteins from other species in National Center for Biotechnology Information (NCBI) database. PAX1 Protein sequence reference number in NCBI: Human isoform 1 (NP_006183.2); isoform 2 (NP_001244025.1); Chick (NP_001383596.1); Quail (XP_015705637.2); *Xenopus* (XP_002939483.2); Zebrafish Pax1a (NP_001074061.1); Zebrafish Pax1b (XP_700877.6); Medaka (NP_001165520.1).

PAX1 localizes to chromosome 2 in mouse and chromosome 20 in human (Chalepakis et al., 1991; Stapleton et al., 1993). PAX1 contains a 128-amino acid paired-box domain (PD) which is responsible for DNA-binding and an 8-amino acid octapeptide domain (OP), which exhibit extremely high levels of sequence identity among different species (Figure 1). Both PD and OP are involved in protein-protein interactions as reported in the PAX family. For example, PD in PAX3 is essential for its interaction with SOX10 (Lang and Epstein, 2003), while OP is required for the interactions between PAX5 and GRG4 (Eberhard et al., 2000).

PAX1 is essential for the development of multiple tissues during embryogenesis, such as thymus (Wallin et al., 1996; Su and Manley, 2000; Su et al., 2001; Yamazaki et al., 2020), vertebral column (Wallin et al., 1994; Smith and Tuan, 1995; Furumoto et al., 1999; Peters et al., 1999; Sivakamasundari et al., 2017), chondrogenic differentiation and chondrocyte maturation (Rodrigo et al., 2003; Takimoto et al., 2013). In this review, we will summarize not only the roles of PAX1 in embryonic development and relevant diseases associated with PAX1 mutations, as well as its molecular modes of action during embryogenesis, but also significant advances in its emerging roles in various kinds of cancers, therefore providing insights for the molecular basis of PAX1 in congenital defects and cancers.

## 2 Pax1 displays conserved expression patterns during vertebrate embryonic development

During mouse embryonic development, Pax1 transcripts can be detected as early as E8.5 in the ventromedial part of newly formed somites when they are undergoing de-epithelialization (Wallin et al., 1994), as well as in the endoderm of the foregut region (Wallin et al., 1996). At E9.5, Pax1 starts to express in the sclerotome cells and becomes stronger at E10.5 in a subset of sclerotome cells which will migrate towards the notochord (Wallin et al., 1994). Meanwhile, Pax1 is also present in the limb buds (Koseki et al., 1993; Timmons et al., 1994), as well as in the first three pharyngeal pouches, which will fuse with ectodermal cells of the third branchial cleft to form the early thymic epithelial primordium and then from E11.0 onwards is populated by lymphoid progenitor cells (Wallin et al., 1996). At E12.5, when sclerotome cells migrated to locate close to the notochord, Pax1 can be detected in the condensation part which contributes to the intervertebral discs (IVDs), but not in the cells giving rise to the vertebral bodies (VBs) anlagen (Deutsch et al., 1988; Wallin et al., 1994). It also appears in the proximal part of the ribs, facial mesenchyme, sternum and pectoral girdle (Koseki et al., 1993; Timmons et al., 1994). In the meantime, Pax1 shows expression in a large number of epithelial cells in the thymus anlagen, that continues to be maintained up to the adult thymus, even though at which stage Pax1 can only be observed in a small number of cortical epithelial cells (Wallin et al., 1996). Till E14.5, Pax1 expression is confined to the anlagen of the IVDs and in a layer of cells in the perichondrium surrounding the VBs anlagen, but not in the ossifying vertebrae (Deutsch et al., 1988; Wallin et al., 1994). Similar observations were shown in quail embryos that Pax1 expression becomes restricted to the IVDs and the perichondrium of the VBs, as well as the connective tissue surrounding the spinal ganglia, after the Pax1-positive sclerotome cells migrated to surround the notochord (Ebensperger 1995). While in chick embryos, it is interesting to note that Pax1 not only shows expression in the IVDs, but also in the chondrocytes of immature vertebral bodies, so far unreported for mouse Pax1 (Peters et al., 1995). *In situ* hybridization and Northern blot hybridization showed that Pax1 is also expressed in the limb buds and pharyngeal pouches in developing chick embryos and continuously to be detected in the adult thymus (Peters et al., 1995). In *Xenopus* embryos, Pax1 transcripts starts to be detected in early somitogenesis (st. 17) and becomes more and more abundant (st. 20–45) in the sclerotome and endodermal pharyngeal pouches, as reported for other vertebrates (Sanchez and Sanchez, 2013). Zebrafish has two pax1 paralogues (pax1a and pax1b), both of which exhibit expression in the developing pharyngeal pouches, and sclerotomes from 18 to 96 hpf (Liu Y. H. et al., 2020).

Altogether, the expression profiles of Pax1 in vertebrates show a highly restricted pattern and are mostly comparable to each other in different species, which might be due to the highly conserved sequences of Pax1 (Figure 1).

## 3 Biological functions of Pax1 in embryonic development

In vertebrates, spontaneous mutants and genetic approaches help to reveal the biological functions of Pax1 in embryonic development. In accordance with its expression profile, Pax1 is essential for the development of axial skeleton, limb bud and pectoral girdle, as well as pharyngeal pouches-derived tissues, especially thymus (Chalepakis et al., 1991; Timmons et al., 1994; Wallin et al., 1994; Furumoto et al., 1999; Peters et al., 1999; Su and Manley, 2000; Su et al., 2001; Aubin et al., 2002; Sivakamasundari et al., 2017; Yamazaki et al., 2020).

### 3.1 The roles of Pax1 in skeleton development.

An allelic series of spontaneous mouse mutants have been reported and are important for understanding the critical roles of Pax1 in the development of skeleton system (Chalepakis et al., 1991; Timmons et al., 1994; Wallin et al., 1994; Dietrich and Gruss, 1995). *Undulated* (*un*) mice, carrying a point mutation with a Gly-Ser exchange at position 15 in the conserved part of the paired-box domain of Pax1, which dramatically decreases the DNA-binding affinity and alters the DNA-binding specificity of Pax1, showed kinked tails and skeletal deformity, suggesting a role of Pax1 in the skeleton formation (Wright, 1947; Chalepakis et al., 1991). *Undulated extensive* (*un-ex*) mice carry a deletion of at least 28.2 kb, removing the terminal Pax1 exon including the poly A signal, leading to a drastically reduced amount of Pax1 transcripts (Dietrich and Gruss, 1995). It showed severe skeletal malformations in homozygous animals, and only occasional mild skeletal abnormalities have been described in heterozygotes, similar with *un* mutant. Thus, *un-ex* and *un* are regarded to be recessive (Wallin et al., 1994; Wilm et al., 1998). Whereas, *undulated short tail* (*un-s*), is semidominant as heterozygotes exhibit clear skeletal abnormalities including a very short and strongly kinked tail (Wallin et al., 1994). Homozygous Pax1 *un-s* mice die perinatally displaying the most severe skeletal malformations among the *undulated* alleles (Wallin et al., 1994; Wilm et al., 1998). This can be explained by later findings of the molecular basis of *un-s* mutant mouse that harbors a Pax1 deletion interval of 125 kb, affecting four physically linked genes within or near the deletion, including Pax1, Nkx2-2, and their potential antisense genes (Kokubu et al., 2003). Moreover, another spontaneous mutant mouse, called *scoliosis* (*sco*), carries a new allele of Pax1 (*un-i*, *undulated intermediate*). The Pax1*un-i* allele is lacking the 5′-flanking region and exon 1 to 4 which is mapped to nt −2636 to 640 and −272 to 4271 of the Pax1 gene. Homozygous *sco* mice show a mild form of the known phenotypes of other Pax1 mutants and have a lumbar scoliosis and kinky tails in adults (Adham et al., 2005). In addition, the proximal part of the ribs are missing or severely malformed in Pax1 mutant mice (Wallin et al., 1994), suggesting an essential role of Pax1 for the development of the proximal part of the ribs. Moreover, Pax1 null mice generated by gene targeting showed strong skeletal abnormalities all along the vertebral column, in the sternum, and in the scapula, similar as those found in the mutant mice, with phenotypic differences in degrees of severity (Wilm et al., 1998), suggesting its roles in these tissues in accordance with its expression profile.

**FIGURE 2:**
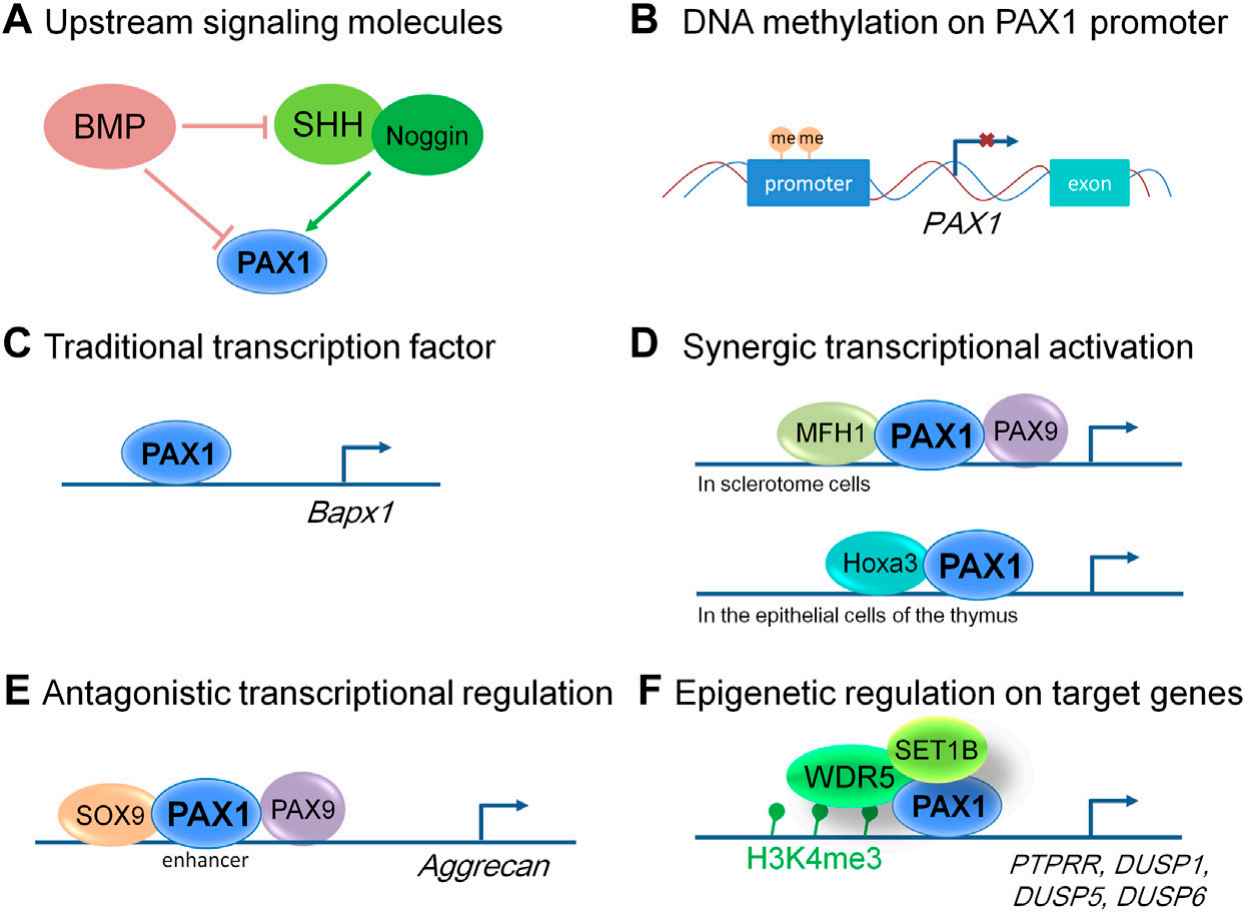
Molecular modes of action of PAX1 and its up and downstream events. **(A)** Signaling molecules function upstream of PAX1 in embryonic development. **(B)** PAX1 is hypermethylated at its promoter region in many kinds of cancer cells, leading to decreased level of PAX1 expression. **(C)** PAX1 can function as a traditional transcription factor to transactivate regulatory sequences in the *Bapx1* promoter region in sclerotome cells. **(D)** PAX1 acts on cell proliferation synergistically with MFH1 and PAX9 in sclerotome cells and Hoxa3 in the epithelial cells of the thymus. **(E)** PAX1 and PAX9 play antagonistic roles with SOX9 in the enhancer region of *Aggrecan* in the developing vertebral column. **(F)** PAX1 forms a complex with WDR5 and SET1B to regulate the H3K4me3 level within the promoters of *PTPRR*, *DUSP1*, *DUSP5*, and *DUSP6*.

In chick embryos, injection of an antisense oligodeoxynucleotide (ODN) against Pax1 resulting in decreased expression of Pax1 transcript leads to the loss of somite and/or disordered somite phenotype, implying the importance of Pax1 in proper segmentation of the somites and in sclerotomal differentiation (Smith and Tuan, 1995; Hol et al., 1996). Pax1 not only functions in the axial patterning, but also is involved in the formation of appendicular skeleton in chick embryos. Embryonic chick wings show morphological defects in the shoulder girdle when Pax1 expression is reduced by ectopic application of signaling molecules (BMP2 and BMP4) (Hofmann et al., 1998), which parallel the defects seen in Pax1 mutant mice (Timmons et al., 1994; Dietrich and Gruss, 1995). In zebrafish, microinjection of morpholino- (MO-) modified antisense oligonucleotides against *pax1b* induced pectoral fin bud defects which could not be phenocopied by *pax9* MO and could not be rescued by either *pax1a* or *pax9* overexpression, whereas could be partially rescued by mouse *Pax1* mRNA injection (Liu et al., 2013; Chen et al., 2014). Loss-of-function of *pax1b* in zebrafish affects the expression of *col2a1*, *uncx4.1*, *noggin3* and *aggrecan*, suggesting its roles in chondrocytes differentiation (Chen et al., 2014). While in chick embryos, Pax1 acts synergistically with Pax9, mediating Shh signaling from the notochord and the floor plate, and directly induces *Bapx1* (also known as *Nkx3.2*) expression in chondrogenic differentiation of sclerotomal cells (Rodrigo et al., 2003) (Figure 2). Whereas, as cartilage formation proceeds, overexpression of Pax1 in chick embryos inhibits chondrocyte maturation, but does not affect mesenchymal condensation prior to cartilage formation and the subsequent early differentiation processes to give rise to proliferating chondrocytes (Takimoto et al., 2013). Cell culture studies showed forced expression of Pax1 in chondrocytes induced a morphological change from polygonal to fibroblastic, significant decrease in proteoglycans (PGs) accumulation, and downregulation of cartilage marker genes including *Chm1*, *Col2a1*, *Aggrecan*, *Ihh*, *Nkx3.2* and *Sox9*, which can be partially rescued by Sox9 overexpression, suggesting Pax1 antagonizes the Sox9-driven chondrocyte maturation to act as a negative regulator during chondrogenic differentiation (Takimoto et al., 2013). This is in agreement with the study that PAX1/9 competes with SOX9 for occupancy of the binding site on the enhancer of *Aggrecan*, resulting in its reduced transactivation in the annulus fibrosus (AF) of IVDs (Takimoto et al., 2019). Consider that the expression of Pax1 becomes downregulated and restricted to the mesenchymal condensations that give rise to the anlagen of the IVDs, and to the perichondrium surrounding the cartilaginous VBs in physiological conditions (Wallin et al., 1994), supporting the notion that Pax1 plays multifaceted roles during the vertebral formation in a spatiotemporal and context-dependent manner.

Using double mutant animal models, PAX1 was shown to act cooperatively with its cofactors PAX9 and MFH1 (also known as FOXC2) to regulate the proliferation of sclerotome cells during vertebral column formation (Furumoto et al., 1999; Peters et al., 1999), and cooperates with Hoxa5 in the development of the pectoral girdle (Aubin et al., 2002; Figure 2). Pax1/Pax9 double mutant mice completely lack the medial derivatives of the sclerotomes, the VBs, IVDs and the proximal parts of the ribs, which is much more severe than that of Pax1 single homozygous mutants (Peters et al., 1999). Whereas formation and anteroposterior polarity of sclerotomes, as well as induction of a chondrocyte-specific cell lineage, appear not affected (Peters et al., 1999). Reduced cell proliferation in the ventromedial regions of the sclerotomes was observed after sclerotome formation (Peters et al., 1999), implying that PAX1 works in concert with PAX9 in the migration, proliferation and mesenchymal condensations of the sclerotomes, but not in the earlier processes of somitogenesis. A similar effect was observed for MFH1 and PAX1. The differentiation of somites into sclerotome, myotome, and dermatome occurs in Mfh1/Pax1 double mutants, indicating that MFH1 and PAX1 are not essential for sclerotome formation (Furumoto et al., 1999). They function synergistically in the regulation of proliferation or migration of sclerotome cells toward the notochord, resulting in more severe malformation of the vertebral column in Mfh1/Pax1 double mutant animals (Furumoto et al., 1999).

As a transcription factor, PAX1 is able to bind to the promoter region and regulate the expression of its downstream target genes. However, there are few direct target genes of PAX1 uncovered so far. There is no doubt that emerging molecular technologies will help in discovery of PAX1 target genes and revealing of the molecular mechanisms underlying its roles in skeleton development. Recently, more PAX1 downstream targets were found by a microarray in mouse vertebral column tissue, such as *Wwp2*, *Col2a1* and *Hip1*, which are cooperatively regulated and occupied by PAX9 in mouse IVDs of the axial skeleton as shown by chromatin immunoprecipitation sequencing (ChIP-Seq) (Sivakamasundari et al., 2017). However, no evidence showed whether they are directly bound by PAX1 due to lacking of a good ChIP-grade antibody against PAX1 in the study (Sivakamasundari et al., 2017).

Moreover, Shh signals from the notochord are necessary for the formation of the sclerotome from the epithelial somite which does not require PAX1, but the induction of PAX1 expression by Shh signals is essential for the specification of the sclerotome cells to obtain ventral or dorsal characteristics (Koseki et al., 1993; Furumoto et al., 1999). Noggin, which encodes a BMP antagonist, was required for Shh-mediated induction of sclerotomal development either by activating PAX1 alone or acting synergistically with Shh (Macmahon 1998), whereas BMP2 and BMP4 proteins supplied by the paraxial mesoderm itself, or by adjacent tissue completely abolished PAX1 induction in response to Noggin, Shh, or their combination (Macmahon 1998) (Figure 2). Overall, the signaling molecules cross talk with each other within the sclerotome cells and with tissues nearby, playing a role in PAX1 expression regulation and form a regulatory network together to control skeleton formation.

In summary, PAX1 does not function in the formation of the sclerotome from epithelial somite, but is essential for the specification, proliferation, migration, as well as differentiation of sclerotome cells in the following stages after sclerotome formed, thus plays critical roles in the development of the skeleton system. We summarized the reported molecular modes of action of PAX1 in Figure 2. But the regulations of PAX1 are far from clear. For example, questions still remain that the regulation of PAX1 at transcriptional and post-translational levels are almost lacking. Answering these questions will not only help us to understand the biology of PAX1 better, but may also reveal novel diagnostic approaches that we can use in PAX1-associated diseases.

### 3.2 The roles of Pax1 in thymus development

The roles of Pax1 in thymus development have also been extensively investigated, since Pax1 is highly expressed in the third pharyngeal pouch, which gives rise to the thymus epithelium (Wallin et al., 1996). Mutations in Pax1 gene in *un*, *un-ex*, *un-s* mice all affected not only the total size of the thymus but also the maturation of thymocytes (Wallin et al., 1996). Further studies clarified that mutations in Hoxa3 and Pax1 act synergistically to cause defective thymic epithelial cell development, resulting in thymic ectopia and hypoplasia (Su and Manley, 2000), due to increased death and decreased proliferation of thymic epithelial cells caused by altered Hoxa3-Pax1 genetic pathway during thymus organogenesis (Su et al., 2001). Importantly, PAX1 mutants (Cys368*; Asn155del and Val147Leu) were identified in otofaciocervical syndrome type 2 (OTFCS) patients with severe combined immunodeficiency (SCID), which characterized by severe T cell lymphopenia, causing increased susceptibility to viral, bacterial, and fungal infections since early in life, failed to attain T cell reconstitution after allogeneic hematopoietic stem cell transplantation (Yamazaki et al., 2020). It showed an altered conformation and flexibility of the paired-box domain and reduced transcriptional activity of PAX1 (Yamazaki et al., 2020), which lead to an altered transcriptional profile in iPS (induced pluripotent stem cells) derived thymic epithelial progenitor cells, suggesting an essential role of PAX1 in human thymus development and function as well (Yamazaki et al., 2020).

## 4 Mutations and dysregulation of PAX1 in diseases

### 4.1 Human syndromes associated with PAX1 mutations

Consistent to its function in axial skeleton development, PAX1 coding variants were identified in the heterozygous state in exon 4 in two male patients with congenital vertebral malformations (Giampietro et al., 2005). One patient who showed thoracic vertebral segments T9 hypoplasia, T12 hemivertebrae and absent T10 pedicle, incomplete fusion of T7 posterior elements, ventricular septal defect, and polydactyly, had CCC (Pro) to CTC (Leu) substitution at amino acid 410, while the other patient had a T11 wedge vertebra and a missense mutation at amino acid 413 corresponding to CCA (Pro) to CTA (Leu) (Giampietro et al., 2005) (Figure 1). Since no molecular studies shown in the report, whether these two PAX1 mutations are responsible for the phenotypes shown in the patients need further investigations. PAX1 mutations were also detected in 8 out of 63 patients with Klippel-Feil syndrome (KFS), which is a human congenital condition, characterized by failed segmentation of the cervical vertebrae with the clinical sequelae of a short, immobile neck and a low posterior hairline (McGaughran et al., 2003). Among the 8 cases, three were missense mutations; two patients had intronic changes and three of them had silent mutations. The missense mutations of PAX1 (Pro61Ala; Ala283Pro; Gly289Ser) (Figure 1) could potentially have a pathogenic role, and the question remains whether PAX1 alone, or in conjunction with other genetic or environmental factors, plays a role in the pathogenesis of KFS, need to be confirmed by functional and molecular mechanism studies. These cases are reminiscent of the haploinsufficient roles of PAX1 in the development of skeleton system (Wilm et al., 1998). Moreover, two cases of Jarcho-Levin syndrome (JLS), with severe developmental alterations in the thoracic and vertebral skeleton, including “crab-like” thorax, typical clinical phenotypes of JLS, which have been revealed to stem from dysmorphogenetic reaction of the blastogenic axial skeleton developmental field, showed a significant reduction in protein levels of PAX1 and PAX9 in chondrocytes of the vertebral column (Bannykh et al., 2003). This report implies for the etiology and pathogenesis of JLS, in accordance with the synergistic roles of PAX1 and PAX9 in vertebral column development and chondrogenic differentiation of sclerotomal cells (Peters et al., 1999; Rodrigo et al., 2003). Though the report showed altered expression of PAX1 and PAX9 in JLS, the possibility remains that mutations or dysfunctions of the upstream factors of PAX1 and PAX9 might be involved in the pathogenesis of JLS. Moreover, a PAX1 missense mutation with changing of Gln to His at position 42 in the paired-box domain (Gln139His) was identified in one patient with spina bifida, a kind of congenital malformation due to the neural tube defect (NTD) (Hol et al., 1996). This reminiscent of the phenotypes with a high incidence of lumbar spina bifida in the mouse model crossed of *un* mutant and *Patch* (*Ph*) mutant mice (Helwig et al., 1995) which is a deletion of the gene encoding the platelet-derived growth factor receptor alpha subunit (*PDGFRα*) (Stephenson et al., 1991), suggesting the possibility of an involvement of PAX1 in the occurrence of NTD. Interestingly, PAX1 was shown to regulate the transcriptional activity of *PDGFRα* gene by a luciferase reporter assay, however, additional studies need to be done to indicate whether PAX1 actually binds to the *PDGFRα* promoter directly, or rather mediates its effect *via* interaction with other components of the transcription machinery (Joosten et al., 1998). Since mutation of PAX1 itself is not sufficient to cause NTD as shown in PAX1 mutant animals, the genetic interaction between PAX1 and PDGFR*α,* together with signaling cross talk between the sclerotome cells and the axial tissues that contribute to the neurulation, could increase the risk of NTD formation.

In addition, whole-exome sequencing (WES) of a single pooled DNA sample of four affected individuals in a large consanguineous family with otofaciocervical syndrome (OTFCS) from Turkey, with cup-shaped ears, bilateral mixed hearing loss, bilateral preauricular fistulas, lacrimal duct abnormalities, protruding shoulders, and winged scapulae, identified a homozygous variant (c.497G>T), substituted the glycine at position 166 to valine (p.G166V) within the highly conserved paired-box domain (Figure 1), leading to a significantly reduced transactivation activity of PAX1 (Pohl et al., 2013). Later on, a nonsense homozygous mutation (c.1104C>A, p. Cys368*) and a homozygous small insertion (c.1173_1174insGCCCG, p. Pro392Alafs*19) in PAX1 gene were also identified in OTFCS patients (Paganini et al., 2017; Patil et al., 2018). The major clinical features of OTFCS include ear malformations, facial dysmorphism, shoulder girdle abnormalities, vertebral anomalies, and mild intellectual disability (Patil et al., 2018). Interestingly, some PAX1 mutations (Cys368*; Asn155del and Val147Leu) (Figure 1) found in OTFCS patients accompanied with severe combined immunodeficiency (SCID), who exhibited not only dysmorphic facial features, malformed vertebral bodies and appendicular structures, but also thymus aplasia, showed reduced transcriptional activity of PAX1 on its target gene (Paganini et al., 2017; Yamazaki et al., 2020). These reports confirmed the association of PAX1 gene with facial morphology (Adhikari et al., 2016; Qian et al., 2021), and agree with the roles of PAX1 in the extravertebral structures observed in mice, including the sternum, the scapula and the facial skull (Dietrich and Gruss, 1995), as well as its functions in thymus development (Wallin et al., 1996).

Altogether, almost all the human syndromes associated with PAX1 mutations identified so far are associated with the functions and molecular roles of PAX1 in corresponding tissues reported in animal models, revealing the conserved roles of PAX1 among different species. Thus, the studies in animal models could help people to understand the pathogenesis of human diseases and aid to develop new therapeutic strategies.

### 4.2 The emerging roles of PAX1 in cancer cells

Epigenetic modifications have long been tightly connected with cancer (Dawson and Kouzarides, 2012). DNA methylation catalyzed by DNA methyltransferases (DNMTs) is one of the essential epigenetic modifications that control cell proliferation, apoptosis, differentiation, cell cycle, and transformation in eukaryotes (Pan et al., 2018). Typically, DNA methylation generates a stable epigenetic mark associated with silencing of gene expression (Moore et al., 2012). The genome-wide landscape of DNA methylation in cancer cells is largely altered compared with normal cells (Nishiyama and Nakanishi, 2021). In particular, the early findings have unraveled that aberrant DNA methylation promotes cellular oncogenesis through silencing of tumor suppressor genes (Nishiyama and Nakanishi, 2021).

In the last decade, a growing body of evidence has implied an emerging tumor suppressing role of PAX1 in various human cancers, including cervical cancer (Huang et al., 2010; Kan et al., 2014; Lai et al., 2014; Liu H. et al., 2020; Li et al., 2021), ovarian cancer (Hassan et al., 2017), colorectal carcinoma (Huang et al., 2017), parathyroid tumor (Singh et al., 2022), as well as oral squamous cell carcinoma (Huang et al., 2014; Cheng et al., 2018; Sun et al., 2020), and so on. Higher DNA methylation levels of PAX1 were observed in most kinds of cancer cells (Lai et al., 2008; Huang et al., 2010; Cheng et al., 2016; Huang et al., 2016; Huang et al., 2017; Su et al., 2019; Tang et al., 2019; Zhao et al., 2020; Singh et al., 2022), significantly strengthened the observation that PAX1 often acts as a tumor suppressor. This is a little bit surprising, since PAX1 is only highly expressed in the early embryonic stages while its expression becomes undetectable or very low in the majority of adult human tissues according to the Genotype-Tissue Expression (GTEx) database (Thompson et al., 2021), which is consistent with the observation in mouse model (Deutsch et al., 1988). One possibility could be reactivation of PAX1 occurs under certain circumstances such as drug or stress-induced conditions. PAX1 reexpression was observed in cervical cancer cell lines after treatment with curcumin and resveratrol, may be due to their effect on histone deacetylase mediated through downregulation of UHRF1 which can regulate both DNA methylation and histone acetylation (Parashar and Capalash, 2016). Reactivation of PAX1 due to promoter hypomethylation has been achieved through silencing of DNMT1 in Hela and Siha cell lines (Zhang et al., 2011), suggesting DNMT1 might be the methytransferase responsible for the hypermethylation level of PAX1 in cervical cancer cells.

So far, most reports of PAX1 in cancer cells focus in the methylation status of PAX1, limited articles showed further molecular mechanisms in these processes. Recently, a report showed that PAX1 plays a tumor suppressing role by forming a complex with WDR5 and SET1B, leading to increased trimethylation of histone H3, lysine 4 (H3K4me3), thus activates the expression of multiple phosphatases including *PTPRR*, *DUSP1*, *DUSP5*, and *DUSP6* in cervical cancer cells (Figure 2), maintaining the homeostasis between kinases and phosphatases in cervical epithelium, revealed a functional relevance of PAX1 in cancer biology (Su et al., 2019).

Epigenetic changes are recognized to occur in the early stage of tumor progress and in advanced of genetic alterations, thereby affording a reason for developing biomarkers for early identification and prevention of cancer (Reis et al., 2016). Therefore, PAX1 methylation level not only can provide biomarkers for early detection and diagnosis in cancer patients, but also could predict and monitor early therapeutic response (Pan et al., 2018; Li et al., 2021).

Given its roles in the development of multiple tissues, PAX1 might regulate cell proliferation and differentiation of specific cancer cells and thus contributes to the activation or suppression of cancer development in specific tissue contexts when being dysregulated. As a transcription factor, PAX1 could also drive the suppression or activation of its downstream target genes and participate in the regulation of signaling pathways, or play a role in the epigenetic regulation on its target genes, which may further facilitate or inhibit tumorigenesis in a context-dependent manner.

## 5 Discussion

PAX1 is a highly conserved gene identified early in embryonic development. Its conserved roles can be demonstrated by similar observations in different species. For example, Pax1 is essential for fin bud development in zebrafish, wing bud development in chick and limb bud development in mouse embryos (Timmons et al., 1994; Hofmann et al., 1998; Chen et al., 2014). Interestingly, as a transcription factor in the same subfamily with similar protein domains, PAX9 was often investigated in parallel with PAX1. Undoubtedly, PAX1 and PAX9 play synergistic and redundant roles in some tissues, such as the axial skeleton (Balling et al., 1996; Peters et al., 1999; Sivakamasundari et al., 2017), while they also have their own specific expression and functional tissues as well (Chen et al., 2014; Sivakamasundari et al., 2018). It was found that PAX9 was unable to fully compensate for the loss of PAX1 but PAX1 could fully rescue PAX9 deficiency during the vertebral column formation probably by its own expression level *via* a positive auto-feedback mechanism, suggesting PAX1 is the more dominant player in the axial skeleton development (Peters et al., 1999; Sivakamasundari et al., 2017). Whereas, PAX9 plays essential roles in the palatogenesis and odontogenesis in which PAX1 is largely absent (Peters and Balling, 1998; Kist et al., 2005; Zhou et al., 2013; Jia et al., 2020a; Jia et al., 2020b). In addition, PAX9 expression is not affected when PAX1 is mutated suggesting the expression of PAX9 is not dependent on PAX1 (Neubüser et al., 1995; Sivakamasundari et al., 2017). More interestingly, PAX1 may act downstream of PAX9 in the expanding taste progenitor field of the mouse circumvallate papilla (Kist et al., 2014). Altogether, further studies will be needed to address the clearer relationships between PAX1 and PAX9 in a tissue-specific level.

Even though the expression and functions of PAX1 have been elaborated in details, the cellular and molecular mechanisms that underlie its roles in multiple tissues are far from well-defined. One possibility may be no good commercial PAX1 antibody for biochemical and molecular studies; another reason could be knocking down or knocking out PAX1 in the molecular level is not efficient enough with common used methods such as siRNA mediated gene silencing techniques. With the development of the state-of-the-art techniques and the implement of emerging powerful tools of molecular and genetic studies such as high-throughput sequencing and CRISPR-Cas9 genome editing techniques, more and more PAX1 interacting partners and PAX1 direct targets in different tissues could be brought to light. Furthermore, the integrating of PAX1 activities to the regulatory network orchestrating embryonic development and diseases is a far-reaching task which will help us to see the whole picture and provide clinical implications in the future.
